# *In situ* degradation of antibiotic residues in medical intravenous infusion bottles using high energy electron beam irradiation

**DOI:** 10.1038/srep39928

**Published:** 2017-01-03

**Authors:** Min Wang, Lele Zhang, Guilong Zhang, Tao Pang, Xin Zhang, Dongqing Cai, Zhengyan Wu

**Affiliations:** 1 Key Laboratory of Ion Beam Bioengineering, Hefei Institutes of Physical Science, Chinese Academy of Sciences, Hefei 230031, People’s Republic of China; 2 University of Science and Technology of China, Hefei 230026, People’s Republic of China; 3 School of Life Sciences, Anhui Agricultural University, Hefei 230036, People’s Republic of China; 4 Anhui Jianghuai Automobile Co., Ltd, Hefei 230022, People’s Republic of China; 5 Key Laboratory of Environmental Toxicology and Pollution Control Technology of Anhui Province, Hefei Institutes of Physical Science, Chinese Academy of Sciences, Hefei 230031, People’s Republic of China

## Abstract

This study reported an immediate approach for the degradation of three antibiotic (amoxicillin, ofloxacin, and cefradine) residues in medical intravenous infusion bottles (MIIBs) using high energy electron beam (HEEB) irradiation. The effects of irradiation doses, initial concentrations, initial pH, and scavengers of active radicals on the degradation of three antibiotic residues (ARs) were investigated, and the results displayed that 97.02%, 97.61% and 96.87% of amoxicillin, ofloxacin, and cefradine residues could be degraded *in situ* through HEEB irradiation respectively. Fourier transform infrared spectroscopy (FTIR) and high performance liquid chromatography-mass spectrometry (HPLC-MS) analysis demonstrated that ARs were mainly decomposed into inorganic ions and alkanes. Typically, the detailed degradation mechanism of ARs was also investigated, and the dominant active particle inducing the degradation of antibiotics during the HEEB irradiation process was demonstrated to be hydroxyl radical.

Antibiotics have been widely used in the treatment of bacterial infections for both humans and animals. According to previous studies, 100,000 to 200,000 tons of antibiotics are used around the world every year^1^. Hence, plenty of medical intravenous infusion bottles (MIIBs) with antibiotic residues (ARs) were generated and exposed to environment, causing extremely harmful effects on human health (arthropathy, nephropathy, mutagenic effect, damage of central nervous system, growing of antibiotic-resistant bacteria) and ecological balance (damage on the structure of microbial community)^2^,^3^,^4^,^5^,^6^,^7^. Therefore, it is rather important to develop an efficient method to degrade ARs.

In the past a few years, several approaches for degradation of antibiotics have been developed mainly through adsorption, catalytical degradation, and biodegradation^8^,^9^,^10^,^11^. Those could degrade or remove antibiotics to different extents, however they displayed a few disadvantages: high cost and secondary pollution for the former method, low efficiency and strict condition for the latter two methods^12^,^13^,^14^. Recently, high energy electron beam (HEEB) irradiation has attracted more and more attention due to its properties of high efficiency, clean, low cost, and available for batch processing^15^,^16^,^17^. Furthermore, HEEB irradiation can facilely generate highly reactive species in aqueous solution, such as reductive solvated electron (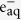), hydrogen atom (H·), and oxidative hydroxyl radical (OH·), which are favor to the degradation of organic pollutants. Therefore, we decided to treat the ARs by HEEB irradiation.

Here, the application of HEEB irradiation on the immediate and *in situ* degradation of ARs in MIIBs has been reported. This work will lower the cost of the treatment of ARs because ARs in MIIBs are easily to be collected and *in situ* degraded. Amoxicillin (AMX), ofloxacin (OFL), and cefradine (CED) are selected as representative antibiotics of β-lactams, cephalosporins, and quinolones, respectively, which have been widely used in human and veterinary medicines for their effective broad spectrum against a wide variety of microorganisms^1^,^3^,^6^,^18^. In the present study, HEEB displayed fine performance on the degradation of ARs in MIIBs. Meanwhile, the effects of initial concentrations of antibiotics, HEEB irradiation doses, pH of initial solutions, and common coexistent substances on AR degradation behavior were studied in detail to obtain the optimal degradation condition, and the reaction mechanism was also investigated.

## Results and Discussion

### Effects of HEEB irradiation dose on the degradation of AR

**Figure 1:**
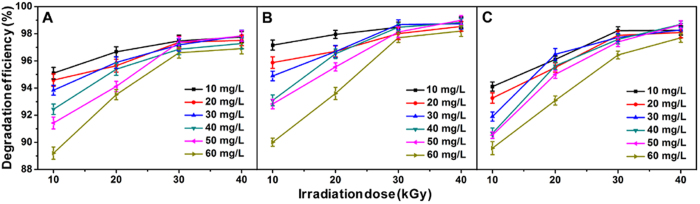
Degradation efficiencies of AMX (**A**), OFL (**B**) and CED (**C**) treated by HEEB irradiation with different doses.

A batch of experiments were conducted to investigate the effects of HEEB irradiation dose and initial concentrations of antibiotics on the DEs of ARs. As shown in Fig. 1, the DEs of ARs increased with HEEB irradiation dose at first (<30 kGy) and became relatively stable afterwards (>30 kGy), which was probably because the amounts of active particles generated in the AR solutions increased with irradiation dose (<30 kGy), and then became almost sufficient for the degradation of all the ARs (>30 kGy). Additionally, when the initial concentration of AR was below 50 mg L^−1^, the DE of each AR almost decreased with the increasing initial AR concentration when the irradiation dose was lower than 20 kGy, and reached nearly same value when the dose was higher than 30 kGy. This was because the ratio (amounts of active particles/amounts of AR molecules) decreased with the increasing initial AR concentrations when the dose was lower than 20 kGy, while the active particles were sufficient for the degradation of almost all the AR molecules with dose higher than 30 kGy. When the initial concentration of AR was 60 mg L^−1^, the DE of AR appeared lower compared with AR with the concentration below 50 mg L^−1^. Therefore, the optimal dose of HEEB irradiation was 30 kGy and the optimal initial concentrations of three ARs were 50 mg L^−1^. Therein, at irradiation dose of 30 kGy, the DE order was OFL > AMX > CED, which was probably attributed to their molecular structures.

### Effect of pH on the degradation of AR

**Figure 2:**
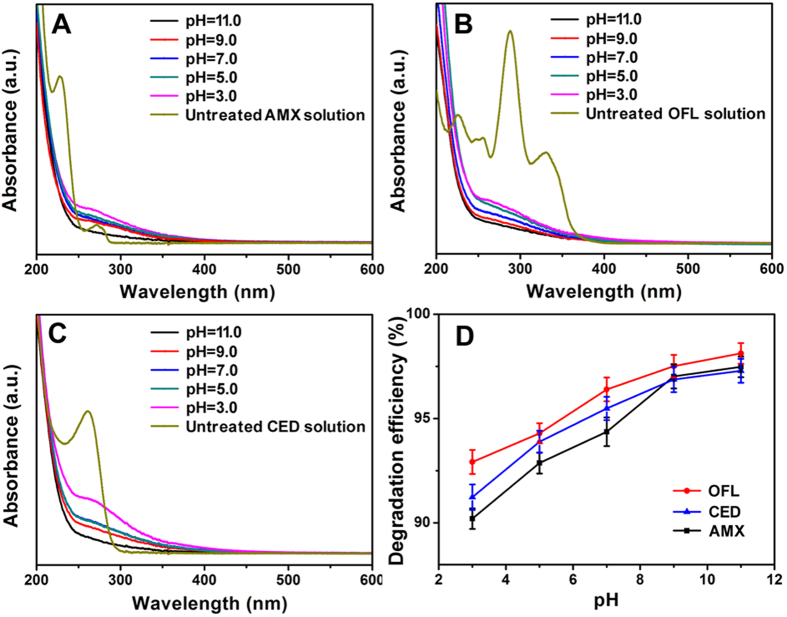
UV absorption spectra of AMX (**A**), OFL (**B**), CED (**C**), and the DEs (**D**) of ARs (50 mg L^−1^) at different pH with irradiation dose of 30 kGy.

The influence of pH on the degradation performance of ARs was also investigated. Fig. 2A–C showed the UV absorption spectra of AMX, OFL, and CED at different pH values respectively. It could be seen that the absorption intensities of AMX, OFL, and CED all decreased with the increasing pH. Accordingly, the DEs of AMX, OFL, and CED increased with pH (Fig. 2D), which was probably because alkaline condition could effectively promote the dissociation of AR molecules and the generation of active particles in the solution, and meanwhile the dissociated AR molecules tended to be degraded by these active particles^19^. Considering the slow increasing trend when pH was higher than 9.0, the pH for the degradation of ARs was selected as 9.0.

### Degradation products investigation

#### FTIR analysis

**Figure 3:**
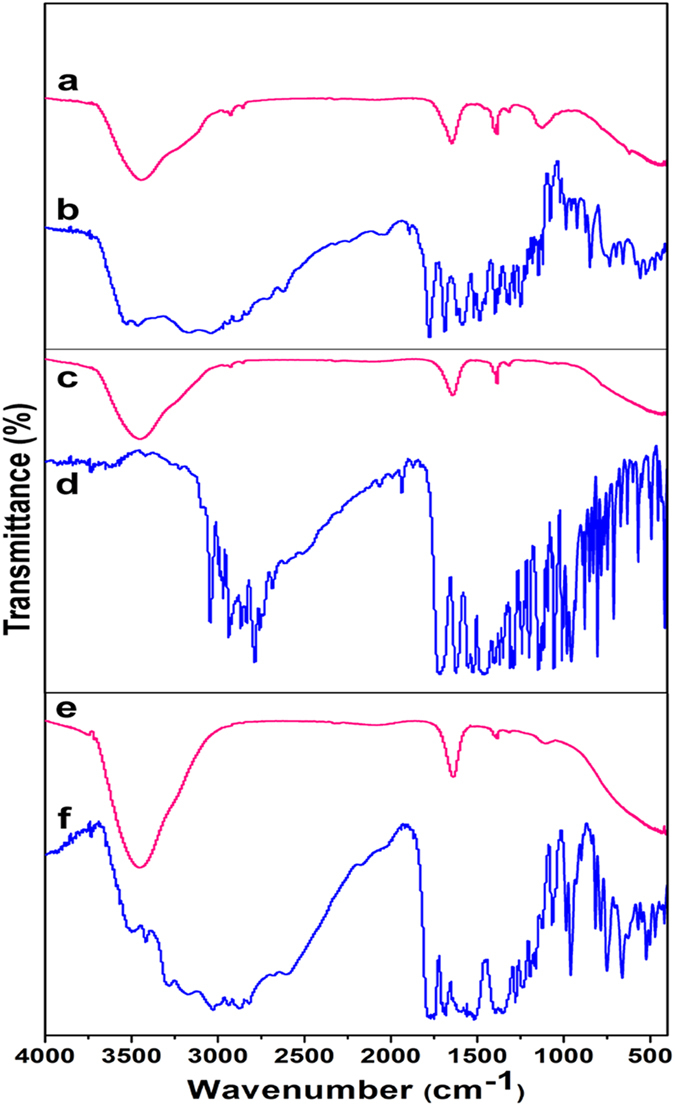
FTIR spectra of samples: (**a**) AMX (50 mg L^−1^) after degradation at dose of 30 kGy with pH of 9.0, (**b**) AMX, (**c**) OFL after degradation at dose of 30 kGy with pH of 9.0, (**d**) OFL (50 mg L^−1^), (**e**) CED after degradation at dose of 300 kGy with pH of 9.0, (**d**) CED (50 mg L^−1^).

FTIR measurement was performed to investigate the degradation products of ARs. Fig. 3b showed the standard spectrum of AMX, the intensified peaks at 1687, 1776, and 3398 cm^−1^ were corresponding to C=O of N-C=O, C=O of HO-C=O, and -OH of Ar-OH, respectively^6^. As shown in Fig. 3d, the absorption peaks at 1622 and 1713 cm^−1^ were ascribed to C=O of -COOH and -C=O- of OFL^20^. Additionally, a series of characteristic absorption peaks at 470, 664, 787, 1606, 1687, and 1773 cm^−1^ of CED were clearly observed in Fig. 3f. Moreover, the FTIR spectra of degradation products of AMX, OFL, and CED after HEEB irradiation exhibited the similar absorption peaks as shown in Fig. 3a,c and e, respectively, suggesting that the degradation products possessed the similar chemical structures. According to previous studies^6^,^20^, the absorption peaks at 3400 cm^−1^ could be assigned to the stretching vibration of -OH, and the peaks at 1450, 2900, and 1360 cm^−1^ confirmed the existence of -CH_2_ and -CH_3_ in the degradation products. Meanwhile, the absorption peaks between 3100 and 3010 cm^−1^ of AMX, OFL, and CED disappeared in the spectra of degradation products, indicating the destruction of unsaturated hydrocarbons in antibiotics. These results indicated that the degradation products of ARs after HEEB irradiation included saturated alkane.

#### HPLC/MS analysis

**Figure 4:**
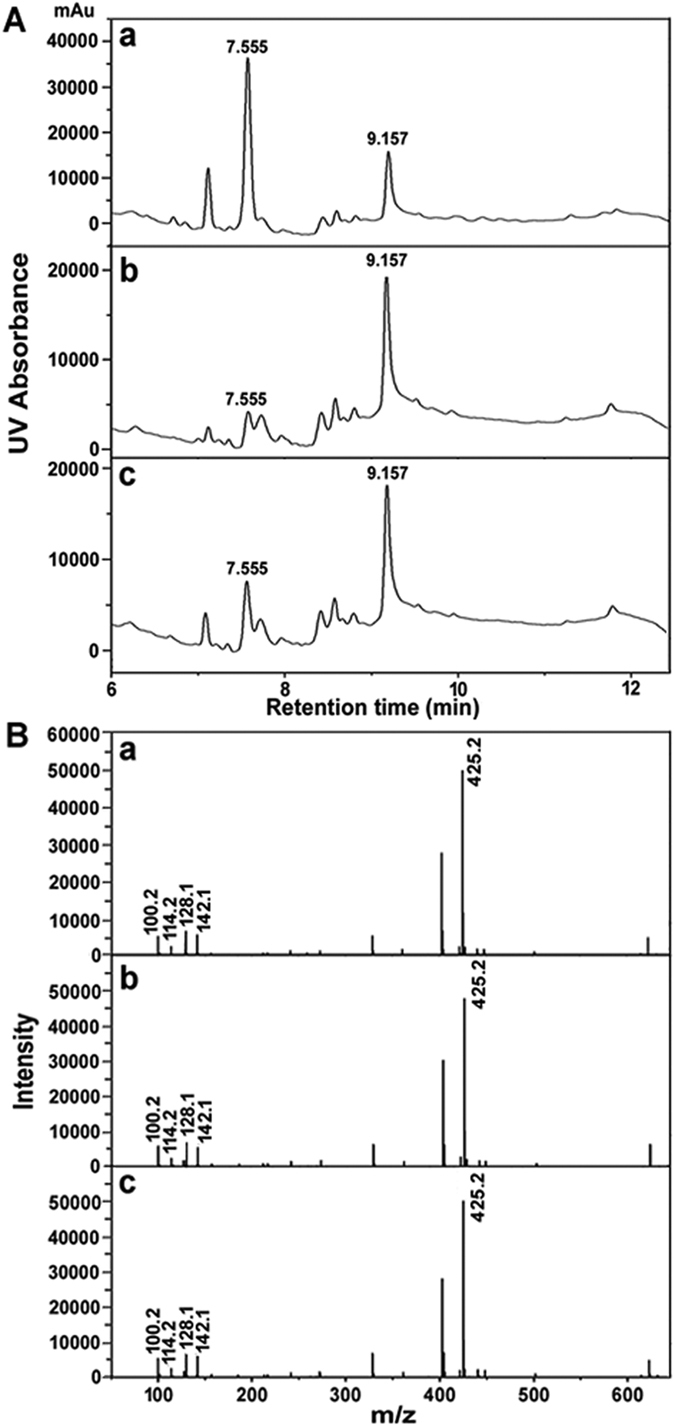
(**A**) HPLC chromatograms of degradation products of AMX (a),OFL (b), and CED (c), (**B**) MS spectra of degradation products of AMX (a), OFL (b), and CED (c) collected at retention time of 9.157 min after HPLC treatment.

HPLC measurement was performed to investigate the degradation products of ARs. It could be seen clearly in Fig. 4Aa–Ac that the main peaks of AMX, OFL, and CED possessed the same retention time, indicating that three antibiotics possessed the same products after degradation. Meanwhile, the products of AMX, OFL, and CED after HPLC measurement at retention time of 9.157 min were analyzed by MS, which showing the similar patterns (Fig. 4B). These results indicated that the degradation products of AMX, OFL, and CED possessed the similar chemical constitutions.

#### IC analysis

According to the previous studies, the organic pollutants could be mineralized into CO_2_, H_2_O, and other inorganic substances by active radicals, and thus the concentrations of inorganic ions in degradation products were determined to evaluate the mineralization degrees of ARs^19^,^21^. As shown in Table 1, the concentrations of 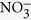, 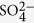 and F^−^ in the degradation products were determined, from which it could be calculated that the average mineralization degrees of AMX, OFL, and CED were 97.02%, 97.61% and 96.87%, respectively. This result demonstrated that the ARs were degraded into inorganic ions including 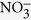, 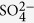, and F^−^ dominantly.

### Mechanism study

HEEB irradiation of water (at pH 3.0–11.0) saturated with N_2_ can generate various reactive species in the aqueous solution as shown in reaction (1)^22^.


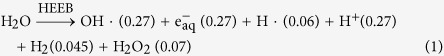


The values in parentheses represent the conversion efficiencies of generated particles during the HEEB irradiation. Among the chemical species formed during the radiolysis of water, OH·, 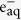 and H· (reactions 1–3) probably displayed activities to different extents for the degradation of organic compounds^20^. In order to evaluate the contribution of each active particle to the degradation of ARs, several scavengers (H_2_O_2_, i-PrOH, and t-BuOH) were used to eliminate these active particles specifically (reactions 4–8): H_2_O_2_ for 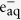 and H·, i-PrOH for H· and OH·, t-BuOH for OH·^15^,^19^,^20^,^21^,^22^,^23^. According to previous studies, the contribution of OH· could be easily assessed in aerated antibiotic solutions, because 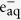 and H· were easily scavenged by oxygen and rapidly converted into HO_2_· (equations 2 and 3)^15^,^19^,^20^,^21^. As shown in reactions (4) and (5), 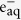 and H· could be oxidized into OH· by H_2_O_2_^23^,^24^.


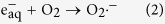



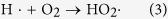



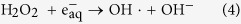



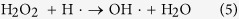



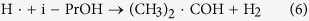



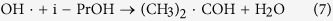



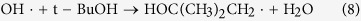


**Figure 5:**
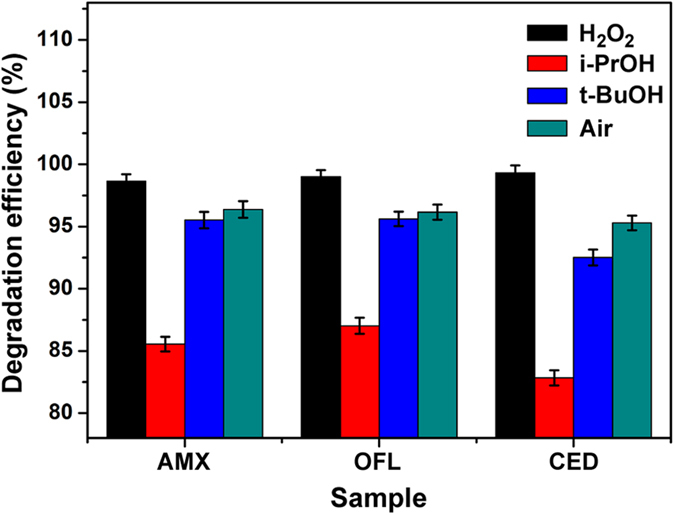
DEs of ARs (50 mg L^−1^) after irradiation (30 kGy) under the conditions of air alone, H_2_O_2_ (0.01 M), i-PrOH (0.01 M), and t-BuOH (0.01 M), respectively.

As shown in Fig. 5, after HEEB irradiation (30 kGy), the DEs of all the ARs with different scavengers (air, H_2_O_2_, i-PrOH, and t-BuOH) displayed the same order (H_2_O_2_ > air > t-BuOH>i-PrOH), which indicated that OH· was probably the dominant active particle for the degradation of ARs, and other active particles such as H· and 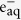 were also responsible for the degradation of ARs in some degree.

#### Effect of coexisting substances on the degradation of ARs

The influences of a series of substances such as nitrite (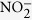), nitrate (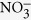), carbonate (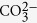), bicarbonate (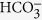), ferric (Fe^3+^), and humic acid (HA) on the degradation of ARs were investigated respectively. These substances except HA tended to react with OH·, H·, and 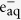 (reactions 9–15) and thus scavenged these radicals to a great extent, while HA could only react with and scavenge OH· according to previous study^20^. It was clearly shown in Table 2 that the DEs of AMX, OFL, and CED with these substances were all lower compared with the corresponding antibiotic alone, suggesting the significant competing effects with antibiotic molecules on OH·^19^,^20^,^21^,^22^,^23^. This result could also prove the key role of OH· in the degradation process of antibiotics.


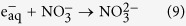



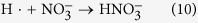



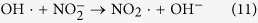



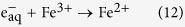



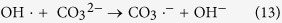



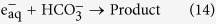



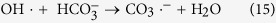


## Conclusions

In summary, three common ARs (AMX, OFL, and CED) in MIIBs were successfully degraded by HEEB irradiation, which could generate plenty of active particles and then effectively degrade ARs *in situ*. It was found that the maximum degradation efficiency was achieved at an initial AR concentration of 50 mg L^−1^ with an irradiation dose of 30 kGy at pH 9.0. FTIR and HPLC-MS analyses illustrated that the degradation products of antibiotics mainly consisted of inorganic ions and alkane. Mechanism analysis indicated that the degradation of ARs was mainly induced by OH· generated during the irradiation process. Additionally, a series of substances coexisting in ARs could affect the degradation performance via reacting with OH·. In a word, this work provided a promising and *in situ* degradation method for ARs.

## Methods

### Materials

AMX (≥99%), OFL (≥99%), CED (≥99%), and terephthalic acid (TA, 98%) were purchased from Aladdin Industrial Co., Ltd. (Shanghai, China). H_2_O_2_, i-PrOH, t-BuOH, HCl, and NaOH with analytical reagent grade were purchased from Sinopharm Chemical Reagent Co., Ltd. (Shanghai, China). Deionized water was used throughout this work. MIIBs (height of 11 cm, bottom diameter of 5.3 cm and volume of 100 mL) made up of propene polymer were obtained from Cancer Hospital, Hefei Institutes of Physical Science, Chinese Academy of Sciences.

### Sample preparation

Antibiotic solutions (50 mL) with concentrations from 10 to 60 mg L^−1^ were prepared to simulate ARs by dissolving AMX, OFL or CED in MIIBs with deionized water. After that, the MIIBs were shaken (100 rpm) for 5 min and then placed vertically for the treatment using HEEB accelerator (10 MeV and 10 kW) with irradiation doses of 10, 20, 30, and 40 kGy, respectively. All experiments were performed at room temperature.

### Degradation performance investigation

The degradation performances of AMX, OFL, and CED were investigated under different reaction conditions. After HEEB irradiation, the MIIBs were shaken (100 rpm) for 5 min and then the concentrations of ARs in MIIBs were determined. The degradation efficiency (DE) was calculated using the following equation:


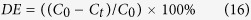


where *C*_*0*_ and *C*_*t*_ are the initial and residual concentrations of antibiotics, respectively^9^,^15^,^18^.

### HPLC/MS analysis

The degradation product analysis was conducted on a mass spectrometer equipped with ESI sources connected to HPLC systems. Analyses were operated in negative ion mode with the mass scanning range of 50–1000 m/z. Isocratic chromatographic elution was performed on Eclipse XDB-C18 column (250 mm × 4.6 mm, 5 μm), using methanol-acetonitrile (1:1, v/v) as a mobile phase. The mobile phase was pumped at a flow rate of 1 mL min^−1^ at room temperature. The injected sample volume was 10 μL. To elucidate the structures of degradation products, HPLC was coupled with a Q-TOF mass spectrometry.

### Mechanism study on the degradation of antibiotics by HEEB irradiation

Before the HEEB irradiation, H_2_O_2_ (0.01 M), i-PrOH (0.01 M) or t-BuOH (0.01 M) was added to the MIIBs containing antibiotic solutions, respectively, and then the solutions were saturated with N_2_^ ^24,25,26. The regular AMX, OFL, and CED solutions were prepared as controls. The resulting MIIBs were shaken (100 rpm) for 5 min and then irradiated by the HEEB accelerator (10 MeV and 10 kW) with dose of 30 kGy at room temperature. Finally, the antibiotic solutions in MIIBs were analyzed to investigate the degradation mechanism.

### Characterizations

The concentrations of antibiotics were determined using a UV-vis spectrophotometer (UV 2550, Shimadzu Co., Japan) at 228.3 nm (AMX), 293 nm (OFL), and 264 nm (CED). The degradation products were analyzed by a Fourier transform infrared (FTIR) spectrometer (Bruker Co., Germany) and a mass spectrometer (MS) equipped with ESI sources (Agilent 6120, USA) connected to high performance liquid chromatography (HPLC) systems (Agilent 1200 infinity, USA). The anions contents in degradation products were analyzed by ion chromatography (IC) (ICS-3000, Dionex Co., USA).

## Additional Information

**How to cite this article**: Wang, M. *et al*. *In situ* degradation of antibiotic residues in medical intravenous infusion bottles using high energy electron beam irradiation. *Sci. Rep.* **7**, 39928; doi: 10.1038/srep39928 (2017).

**Publisher's note:** Springer Nature remains neutral with regard to jurisdictional claims in published maps and institutional affiliations.

## Figures and Tables

**Table 1:** Measured ion concentration (MIC), theoretical ion concentration (TIC), and mineralization degree (MD) of degradation products.

		AMX			OFL			CED	
Sample	MIC (mg L^−1^)	TIC (mg L^−1^)	MD (%)	MIC (mg L^−1^)	TIC (mg L^−1^)	MD (%)	MIC (mg L^−1^)	TIC (mgL^−1^)	MD (%)
F^−^	/	/		2.57	2.63	97.71	/	/	
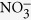	24.69	25.45	97.01	25.12	25.76	97.51	25.79	26.62	96.88
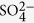	12.75	13.14	97.03	/	/		13.31	13.74	96.87

**Table 2:** DEs of ARs in the presence of substances ( 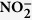 , 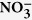 , 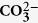 , 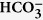 , Fe^3+^, and HA).

Sample	Aerated only	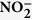	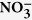	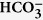	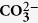	Fe^3+^	HA
AMX	97.52%	80.38%	90.50%	54.92%	80.25%	92.67%	81.96%
OFL	98.91%	77.86%	91.61%	60.14%	76.76%	95.38%	85.72%
CED	98.10%	81.75%	90.76%	61.06%	88.64%	96.29%	83.30%

Experimental conditions: HEEB irradiation dose of 30 kGy, pH = 9.0, [AR] = 50 mg L^−1^, [inorganic ions] = 1M, and [HA] = 35 mg L^−1^.
